# More Than Just Access: Delivering on a Network-Enabled Literature

**DOI:** 10.1371/journal.pbio.1001417

**Published:** 2012-10-23

**Authors:** Cameron Neylon

**Affiliations:** Advocacy Director, PLOS, Cambridge, United Kingdom

## Abstract

To truly gain the benefits of open access, we need to look beyond “access" and ensure that open-access publishing enables re-use, legally and technically, to fully exploit opportunities provided by the worldwide web.

By any measure it has been a huge year for the open-access movement. At the beginning of the year, it looked possible that the public access policy of the US National Institutes of Health (NIH) might be rolled back by the Research Works Act, a legislative attempt supported by Elsevier and the Association of American Publishers to make such policies illegal [1]. But as we move towards year's end, the momentum behind open access looks unstoppable with the announcement of major policy initiatives in the United States, the European Union, Denmark, and the United Kingdom (see Table 1). Nevertheless, there is still much to be done and the challenges remain large, but the remaining questions are largely ones of implementation, not principle.

**Table 1:** Open-access policy initiatives and implementation in 2012.

Country	Policy Initiative
USA	The Federal Research Public Access Act would expand the NIH Public Access Policy to include all US federal agencies with an annual research expenditure of over US$100 million [2].
EU	The Horizon 2020 draft legislation creates a requirement for published research funded by this ∼€80 billion program to be made open access either through a repository or through publication in an open-access journal [3].
UK	The Finch report [4] recommended national adoption of open access for government-funded research. In response, the UK Research Councils have adopted a policy [5] that requires published research to be open access, preferably through journal publication with a CC-BY license. Other UK agencies have also signaled a move towards open-access requirements. The Wellcome Trust also strengthened its open-access policy to require a CC-BY license [6].
Denmark	Five Danish research councils adopted an open-access policy that requires open access through an institutional or disciplinary repository [7].

Each year, a range of open-access organizations support Open Access Week (http://www.openaccessweek.org/), a global event that provides the research community, funding agencies, policy makers, and open-access publishers with an opportunity to discuss, publicize, and advocate for open access. With this year's successes, it is also a good time to reflect on and to consider how we ensure that the promise of open access is delivered. But if we are to exploit the potential that open access provides, we must look beyond just making research findings accessible to ensuring that they are legally and technically available for re-use.

Ten years ago, the authors of the Budapest Open Access Initiative (BOAI) [8] saw this potential clearly and agreed upon a text that has stood the test of time:

By “open access" to this literature, we mean its free availability on the public internet, permitting any users to read, download, copy, distribute, print, search, or link to the full texts of these articles, crawl them for indexing, pass them as data to software, or use them for any other lawful purpose, without financial, legal, or technical barriers other than those inseparable from gaining access to the internet itself. The only constraint on reproduction and distribution, and the only role for copyright in this domain, should be to give authors control over the integrity of their work and the right to be properly acknowledged and cited.

This definition, which could be used to describe the Creative Commons Attribution license (CC-BY) [9], was written nearly a year before the release of the first Creative Commons licenses [10]. PLOS has used CC-BY as its standard license since the publication of its first paper and has supported the principles of full open access throughout its history [11]. In September 2011, 10 years after the original BOAI meeting, the BOAI10 group released a new set of recommendations [12], which explicitly describe the use of CC-BY as best practice. The policies of funding agencies are also increasingly raising the issue of licensing [5],[6]. But why focus on an esoteric legal instrument? Why does licensing matter if we are within sight of making sure that the public can access and read research articles?

Mere access is not enough to deliver on the promise of a truly network-enabled research communication system. The Creative Commons license says that: “You are free: to Share…to Remix…to make commercial use of this work" [9]. This is a “liberal" or “permissive" license, meaning that it does not place any restrictions on the forms of re-use that are allowed. This is consistent with the Budapest Declaration: “The only […] role for copyright […] should be to give authors […] the right to be properly acknowledged […]". Anyone can read the article, but they can also re-use it, in whole or in part for any purpose, including commercial use.

People sometimes struggle with this idea of someone else commercially exploiting their research, and many publishers expressly forbid it. It *is* sometimes difficult to stomach the idea of allowing others to profit from our work, but the reason it is important to enable commercial re-use helps to illustrate a deeper principle about optimizing our use of the web for publishing research. There are at least two realities to the commercialization of academic research. The first is that it is far more likely that someone other than the original research team has the best opportunity to make money out of a specific piece of research. The human genome project generated US$141 for every dollar spent [13], but this immense return is widely distributed as a result of thousands of people's work. It is rare for research teams to have both the academic and business expertise required for commercial exploitation, and they typically require a commercial partner. Open-access publishing effectively increases our chances of finding those potential partners. If we restrict their capacity to use our research by restricting commercial use, we limit the chances of partners, commercial or otherwise, finding and contacting us.

The second reality is that most commercial products are based on multiple pieces of research, not just one. This renders untenable the argument that noncommercial licensing terms are acceptable because a potential commercial user “can always ask". The same argument is often advanced about data sharing. But most people just won't bother asking. In many cases, innovation comes from combining many data sets. It doesn't take too long before it becomes impossible to ask every data holder personally for their permission. Would you want to answer every query? How many could you deal with? One a week? One a day? One an hour?

The whole point of the web is that it exists on a massive scale and the potential value of a network rises as the square of its size [14]. If we want to exploit the web fully for research, we have to think about exploiting its full scale. And that means asking whether the mechanisms we have in place can work at that scale. Personally asking for and granting permission on a case-by-case basis does not.

We need to move from controlling access and permissions to assuring people that they have access and permission with systems that don't require our intervention. The focus should be on enabling and encouraging unexpected uses and re-uses of our research. Making things accessible is a necessary step to make this happen, but it is not sufficient. We must also give potential users the confidence that they have sufficient rights to do what they want. The Creative Commons Attribution license is a good way of achieving this. Even so, providing assurance of the rights to re-use research is still not sufficient.

We also need to ensure that re-use is technically easy. Here there is a significant opportunity to do better. At PLOS we provide all our content in the form of machine-readable XML, but we generally provide figures in flat image formats, reducing the opportunity for data mining. The whole industry could do better by agreeing on standard tag sets and shared systems for the annotation of papers. And we need to do a whole lot better on describing and linking to supplementary data.

When we have these three elements, accessibility, rights, and ease of use, at web scale, then amazing things can happen. When resources and information are embedded in worldwide networks, new kinds of research become possible. Participatory science projects like Fold-It (http://fold.it) and GalaxyZoo (http://www.galaxyzoo.org/) are commonly used examples, but others, such as the explosive growth of bioinformatics as a result of online accessibility of public domain data, are often forgotten. And there are many smaller stories of new connections and projects that have been made possible only because research is easily shareable and useable.

These are really only glimpses of what is possible. The utter transformation of commerce and media that we have seen over the past decade is just starting to take hold in our research work and publishing systems. To realize the full potential, we need to move our agenda beyond merely making things accessible and readable to ensuring that re-use rights are clear, and that technical usability is continuously improving.

And we need to start today. We need to start because the success of open access means that more research is accessible than ever before. It is incumbent on us to show real benefits from that availability. The rapid growth in the number of open-access articles we have seen over the past few years is only going to accelerate as policy initiatives kick in. We will soon have the critical mass of accessible literature that could support the true transformation of scholarly communication.

Mere access is not enough to deliver on the promise of a truly network-enabled research communication system. As open access moves beyond an aspiration to reality, we need to ensure that what is accessible is truly useable, both legally and technically. As we celebrate the gains, and they are real and serious gains [15], let us also keep our eyes on the horizon so that we deliver what we have promised. We need to ensure that licensing is addressed in funder and institutional policies. We need to keep challenging ourselves to improve the technical usability of our publishing platforms and to enable others to experiment. Above all, we need to understand and appreciate what it means to build networked systems that scale to exploit the web. We have promised the world. Now it's time to deliver.
